# ASSOCIATION OF LOWER URINARY TRACT SYMPTOMS WITH INCIDENT FRAILTY AND MORTALITY AMONG OLDER COMMUNITY-DWELLING MEN

**DOI:** 10.1093/geroni/igac059.2115

**Published:** 2022-12-20

**Authors:** Peggy Cawthon, Scott Bauer, Charles E McCulloch, Kristine Ensrud, Kaiwei Lu, Rebecca Scherzer, Kenneth Covinsky, Lynn M Marshall

**Affiliations:** California Pacific Medical Center Research Institute, San Francisco, California, United States; UCSF and San Francisco VA, San Francisco, California, United States; UCSF, San Francisco, California, United States; University of Minnesota Medical School and Minneapolis VA Health Care System, Minneapolis, Minnesota, United States; San Francisco VA, San Francisco, California, United States; San Francisco VA, San Francisco, California, United States; UCSF, San Francisco, California, United States; OHSU-PSU School of Public Health, Portland, Oregon, United States

## Abstract

Lower urinary tract symptoms (LUTS) are associated with increased risk of new mobility and functional limitations among older men. Our objective was to evaluate the longitudinal relationship between baseline LUTS severity and incident frailty or all-cause mortality among 3667 community-dwelling non-frail men age >70 years from the Osteoporotic Fractures in Men (MrOS) study. LUTS severity at analytic baseline was defined using the American Urologic Association Symptom Index (AUASI). Phenotypic frailty was defined at baseline and 2-year follow-up visits using modified Fried criteria and classified as robust (0), intermediate stage (1-2), or frail (3-5); men classified as frail at analytic baseline were excluded. Vital status was assessed every 4 months. Since the proportional odds assumption was not met, we used multivariable multinomial logistic regression to estimate odds ratios (OR) for the association between baseline LUTS severity and incident frailty or death at follow-up compared to robust. OR were adjusted for demographics, health-behaviors, comorbidities, and cognition. After a mean follow-up of 2.3 years, 37% of men were categorized as robust, 46% were intermediate stage, 9.2% developed incident frailty, and 7.9% had died. Per 4 point higher AUASI, the adjusted odds incident frailty versus robust was 1.23 (95% CI 1.12, 1.34). Odds of death versus robust was not statistically significant (OR=1.05, 95% CI 0.94, 1.18). In conclusion, non-frail men with greater LUTS severity at baseline have slightly greater odds of incident frailty within 2 years. Clinicians should be aware that LUTS severity is a prognostic marker for developing frailty in older men.

